# Glioblastoma Mimicking Viral Encephalitis Responds to Acyclovir: A Case Series and Literature Review

**DOI:** 10.3389/fonc.2019.00008

**Published:** 2019-01-22

**Authors:** Keenan Piper, Haidn Foster, Brandon Gabel, Burt Nabors, Charles Cobbs

**Affiliations:** ^1^Ben and Catherine Ivy Center for Advanced Brain Tumor Treatment, Swedish Neuroscience Institute, Seattle, WA, United States; ^2^University of Cincinnati College of Medicine, Cincinnati, OH, United States; ^3^Department of Neurological Surgery, University of California San Diego Medical Center, San Diego, CA, United States; ^4^Department of Neurology, University of Alabama at Birmingham, Birmingham, AL, United States

**Keywords:** glioblastoma, GBM, glioma, encephalitis, viral encephalitis, herpes simplex encephalitis, antiviral, acyclovir

## Abstract

Viral encephalitis and glioblastoma are both relatively rare conditions with poor prognoses. While the clinical and radiographic presentations of these diseases are often distinctly different, viral encephalitis can sometimes masquerade as glioblastoma. Rarely, glioblastoma can also be misdiagnosed as viral encephalitis. In some cases where a high-grade glioma was initially diagnosed as viral encephalitis, antiviral administration has proven effective for relieving early symptoms. We present three cases in which patients presented with symptoms and radiographic findings suggestive of viral encephalitis and experienced dramatic clinical improvement following treatment with acyclovir, only to later be diagnosed with glioblastoma in the region of suspected encephalitis and ultimately succumb to tumor progression.

## Introduction

Viral encephalitis characteristically presents with fever, headache, neurological deficits, and seizures (1). Significant edema or hemorrhage of the temporal lobes as well as regions of hypodensity by T1-weighted magnetic resonance imaging (MRI) are typical (2). Due to the condition's urgency and the fact that evidence of infection can remain absent from cerebrospinal fluid (CSF), presumed herpes simplex encephalitis (HSE) is often treated empirically with acyclovir, a viral DNA replication inhibitor.

While acyclovir is primarily used as a first line defense against herpes simplex virus 1 (HSV-1), it has been shown to be broadly effective against various viruses of the Herpesviridae family, including the β-herpesvirus human cytomegalovirus (HCMV) (3). The presence of HCMV particles in glioblastoma (GBM) tumor tissue of immunocompetent patients was first reported by Cobbs et al. (4). HCMV has since been found to have oncomodulatory properties, including the ability to increase GBM malignancy and stemness both *in vitro* and in murine models, though the virus' presence in GBM has since been the subject of controversy, with multiple groups both corroborating and contradicting its presence. This debate, as well as the myriad documented effects of HCMV on GBM, are reviewed by Foster et al. (5). HCMV localization to glioblastoma cells has been the basis for multiple successful clinical trials, including one showing improved overall survival in glioblastoma patients treated with the antiviral valganciclovir (6, 7) as well as multiple trials of anti-GBM immunotherapy targeting HCMV antigens (8–10).

Glioblastoma has uncommonly been misdiagnosed as viral encephalitis as a result of atypical clinical and radiographic presentation. In this series, we describe three cases in which patients with an initial diagnosis of HSE were treated with acyclovir without steroids, after which all patients showed subsequent symptomatic improvement and ultimately developed a GBM at the site of suspected encephalitis, suggesting either viral implication in the genesis or progression of these patients' tumors or a therapeutic effect of acyclovir on the early signs and symptoms of GBM.

## Background

### Case 1

A 76 year-old male presented with a 3 week history of lightheadedness, olfactory hallucinations, confusion, and intermittent agitation. An MRI was performed, which showed significant edema in the right anteromedial temporal lobe and insula concerning for herpes encephalitis. An electroencephalogram (EEG) revealed a few right frontal sharp waves and diffuse slowing concerning for possible seizure activity. Remarkable laboratory data included a sodium level of 125 mEq/L. CSF revealed a glucose of 62 mg/dL (normal 40–70 mg/dL), total protein of 71 mg/dL (normal 0–44 mg/dL), and 6,750 RBCs with 2 WBCs. CSF testing was negative for human cytomegalovirus (HCMV), herpes simplex virus (HSV), and varicella zoster virus (VZV) by polymerase chain reaction (PCR). CSF was also negative for *Coccidioides* antibodies and cytology for malignant cells.

The patient was started on intravenous (IV) acyclovir for presumed herpes simplex encephalitis and concomitant levetiracetam to mitigate seizure risk. His symptoms improved significantly, and he was discharged on a 21-day course of IV acyclovir. At follow up, roughly 15 days after admission, his prior symptoms of lightheadedness, olfactory hallucinations, confusion, and agitation had all resolved.

A repeat MRI was performed 3 months after symptom onset, showing a ring enhancing lesion concerning for glioblastoma. The patient underwent right temporal craniotomy for resection of the lesion. Pathology was consistent with glioblastoma.

### Case 2

**Figure 1:**
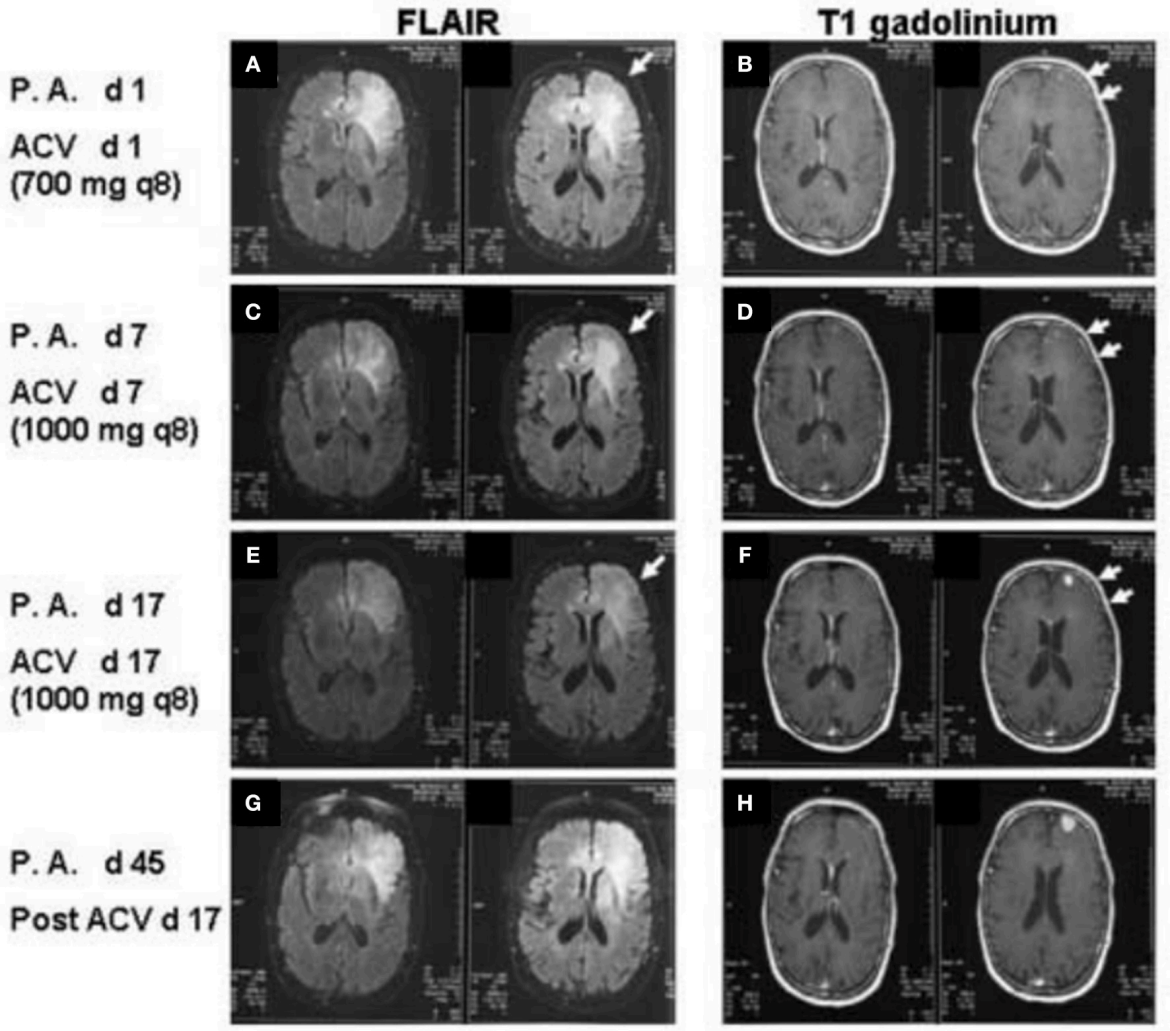
FLAIR images **(A,C,E,F)** T1 post-gadolinium images **(B,D,F,G)** of the patient at increasing days post-admission (P.A. d) and after administration of IV acyclovir (ACV d). Arrows in A, C, and E demonstrate decreasing FLAIR signal in the left frontal white matter from day 1 to day 17 of acyclovir administration. Double arrows in **(B,D,F)** demonstrate decreasing left frontal sulcal effacement and increasing size of the left frontal enhancing lesion. Increasing FLAIR signal and increasing size of the enhancing lesion on T1 post-gadolinium of the left frontal lobe are again noted on post-admission day 45 and post-acyclovir day 17 **(G,H)**.

A 77 year-old male presented with headache, profound confusion, aphasia, and MRI findings of a non-enhancing left frontal lesion which was hyperintense on T2-weighted and FLAIR images (Figures 1A,B). The MRI also revealed non-enhancing lesions in the temporal lobes and corpus callosum. The patient's vital signs on admission were: BP 159/69 mmHg, HR 105 bpm, RR 24, and a temperature of 37.3°C. The patient presented with left carotid bruit. He could not follow commands. His past medical history was significant for hypertension, diabetes mellitus diagnosed 10 years previously, coronary artery disease, and moderately differentiated prostatic adenocarcinoma status post-prostatectomy 10 years previously. Remarkable laboratory data included blood glucose 179 mg/dL and arterial blood gas pH 7.37, pCO_2_ 49, pO_2_ 72, SaO_2_ 94% on 2 L/min O_2_ by nasal cannula.

A neurology consult suggested a possible diagnosis of GBM, but biopsy was deferred due to lack of a ring enhancing lesion. No CSF sample was taken, and IV acyclovir was initiated to treat possible herpes simplex encephalitis. On post-admission day 2, a left internal cerebral arteriogram was performed which demonstrated normal left common, external and internal carotid arteries and normal left anterior and middle cerebral arteries. Acyclovir was administered for 4 weeks. Steroids were not given at any point during the patient's hospitalization. The patient displayed remarkable clinical improvement over the next 2 weeks, with neurological function returning to baseline. MRIs performed on post-admission days 7 and 14 showed decreased edema but interval increase in the size of the focal enhancing lesion along the left frontal lobe gray matter concerning for glioblastoma (Figures 1C–F).

Approximately 3 weeks after his original hospitalization, the patient was readmitted due to neurological deterioration. A fourth MRI scan showed increased enhancement of the left frontal lesion (Figures 1G,H). A fifth MRI, performed ~4 months after his original hospitalization, demonstrated a bifrontal “butterfly glioma.” The tumor was subsequently resected, and pathology confirmed a diagnosis of glioblastoma.

### Case 3

A 78-year-old male presented with severe confusion, receptive aphasia, headache, and dizziness. MRI revealed hyperintensity in the posterior medial left thalamus, bilateral hippocampi, and the left precentral gyrus on T2 FLAIR imaging with no contrast enhancement. Computerized tomography (CT) imaging showed mild microvascular disease but no evidence of acute intracranial process or stenosis. The patient's vital signs upon admission were: BP 103/65 mmHg, HR 64 bpm, RR 18, and a temperature of 36.9°C. His medical history was significant for atrial fibrillation, for which he was prescribed Xarelto but was non-compliant. EEG did not indicate seizure. CSF cultures were negative for HSV and VZV and revealed normal differentiated cell count. Remarkable laboratory results included a CBC with elevated lymphocyte levels of 3.89E9 cells and low creatinine levels of 0.73 mg/dL.

The patient was immediately started on IV acyclovir. No steroids were administered at any point during his hospital stay. The patient displayed clinical improvement and returned to baseline neurological function over the following week.

An MRI performed 2 weeks post-admission revealed stable asymmetric non-enhancing T2 FLAIR hyperintensity involving the left thalamus and increased size of enhancing intra-axial lesion in the left precentral gyrus with surrounding T2 FLAIR hyperintensity, concerning for a neoplastic process. The patient was readmitted 3 months after his initial hospitalization for resection of a brain mass which was determined to be glioblastoma following biopsy.

## Discussion

This report describes three cases of patients with compelling clinical and radiographic evidence of HSE, despite two testing negative by PCR of CSF for common encephalitis-related viruses such as HSV and VZV. All were empirically started on IV acyclovir, regained much or all of their baseline neurological function, and two demonstrated radiographic improvement. Following cessation of acyclovir administration, all three patients experienced a relapse of neurological deterioration and eventual development of glioblastoma at the site of presumed viral encephalitis. Steroids were not administered to any patient during their hospital stay, and no other drugs administered during that period (e.g., levetiracetam) are known to resolve edema.

These findings are consistent with nine other English-language reports describing patients with symptoms of HSE ultimately being diagnosed with GBM or gliomatosis cerebri (11–16). Including the cases presented here, acyclovir was administered empirically in 10 of 12 cases, with only two receiving steroids during their initial hospital stays. Of the ten cases in which acyclovir was administered (Table 1), eight noted clinical improvement following antiviral treatment.

**Table 1:** Cases of glioblastoma masquerading as viral encephalitis.

**References**	**Sex**	**Age**	**Temporal lobe involvement**	**CSF viral PCR**	CSF abnormality^*^	**Acyclovir administered**	**Steroids administered**	**Clinical improvement**	**Radiographic improvement**
This study	M	76	Yes	Neg	Yes	Yes	No	Yes	Unknown
	M	77	Yes	N/A	Unknown	Yes	No	Yes	Yes
	M	78	No	Neg	Yes	Yes	No	Yes	No
Rees and Howard (11)	F	41	Yes	Neg	No	Yes	No	Yes	Unknown
	M	49	Yes	Neg	No	Yes	No	Yes	Yes
	F	72	Yes	Neg	No	Yes	No	No	Unknown
Nam et al. (12)	M	70	Yes	Neg	Yes	Yes	Yes	Yes	Unknown
Wang and Luo (13)	M	50	Yes	Neg	Yes	Yes	Yes	Yes	Yes
Smithson and Larner (14)	M	48	Yes	Neg	No	Yes	No	Yes	Yes
Sun et al. (15)	M	56	Yes	Neg	Yes	Yes	No	No	No

* *Defined as one or more characteristics consistent with viral encephalitis, e.g., moderately elevated protein, elevated mononuclear cell count, and/or moderately decreased glucose*.

Several possible mechanisms of disease are consistent with the atypical progression of glioblastoma and symptomatic responsiveness to acyclovir described in these cases. First, the patients may have developed viral encephalitis and glioblastoma in the same place coincidentally—an unlikely possibility given the relative rarity of these diseases and the time course for their discovery and progression. Alternatively, viral encephalitis could have played a causative role in gliomagenesis. Though most studies suggests that herpesviruses play an oncomodulatory, rather than oncogenic, role in the progression of glioblastoma (17–19), other groups have hypothesized that these viruses may transform cells via a “hit and run” mechanism (20, 21).

One or more patients' tumors may have instead acted as a reservoir for latent infection, which upon reactivation resulted in findings consistent with encephalitis. HCMV, known to latently infect GBM tumors, increases the production of reactive oxygen species and induces expression of cellular enzymes and growth factors, including COX-2 and VEGF, that can contribute to tumor invasion, angiogenesis, and vascular permeability (22). Viral reactivation could therefore result in increased inflammation and tumor malignancy. In this case, it is possible that acyclovir inhibited HCMV-driven phenotypes of infected GBM cells, leading to the observed resolution of initial symptoms.

Finally, glioblastoma may have simply masqueraded as viral encephalitis and responded to treatment with antivirals. Previous studies have shown acyclovir's efficacy in inhibiting *in vitro* growth of glioblastoma cells (23) in addition to its inhibitory effects on regulatory T cells (T-regs) which would otherwise assist glioblastoma in immune evasion (24). Thus, nascent glioblastoma and its associated peritumoral edema may have been responsive to antiviral therapy irrespective of tumor infection. This recalls the non-specific antitumor effects of cidofovir, another antiviral agent which introduces double-stranded cellular DNA breaks leading to GBM oncolysis (25).

## Closing Remarks

Whether via their specific antiviral action or efficacy against GBM directly, antivirals have emerged as a promising therapeutic in the treatment of glioblastoma, though the efficacy of acyclovir in mitigating the early symptoms of GBM tumor progression has yet to be thoroughly studied. Taken in combination with the known inhibitory effects of acyclovir on GBM cells and T-regs, the clinical findings reported here make a strong case for future explorations of adjuvant therapy with acyclovir to treat symptoms of early glioblastoma progression.

## Ethics Statement

The Swedish Medical Center Institutional Review Board determined this case series did not meet the criteria for human subjects research and was not subject to Board review.

## Author Contributions

CC, BG, and BN participated in the patient's treatment. KP, HF, BG, and CC did the literature search and drafted the manuscript. All authors read and approved the final manuscript.

### Conflict of Interest Statement

The authors declare that the research was conducted in the absence of any commercial or financial relationships that could be construed as a potential conflict of interest.

## Abbreviations

CSF: cerebrospinal fluid
CT: computed tomography
EEG: electroencephalogram
GBM: glioblastoma
HCMV: human cytomegalovirus
HSE: herpes simplex encephalitis
HSV: herpes simplex virus
IV: intravenous
MRI: magnetic resonance imaging
PCR: polymerase chain reaction
T-reg: regulatory T-cell
VZV: varicella zoster virus.
